# Cases of Purpura Hæmorrhagica

**Published:** 1826-11

**Authors:** 

**Affiliations:** the Middlesex Hospital.


### PURPURA HÆMORRHAGICA.

*Cases of Purpura Hæmorrhagica.* Treated by Dr. Hawkins, at the Middlesex Hospital.

I. Oct. 30th, 1825.—Sarah Parish, aged fourteen years. Her health has been good until a month ago, when she was attacked with headache and sickness, and the medical man who attended her said that she had slight fever. Her bowels have been kept open by medicine, but she has occasionally vomited a dark matter; her urine and evacuations by stool have also been of a dark colour, but she does not believe that they have contained any blood. About a week ago an oozing of blood commenced from the mouth and nostrils, and has continued ever since. Very soon after the bleeding, purple spots appeared, first on her arms, afterwards on her body and legs, and they have since been increasing in number and size. At present she is covered with petechiæ of a red colour, interspersed with larger, irregular, and purple ecchymoses. Her lips and tongue are covered with sordes and clotted blood. She suffers from thirst, and longs for acid drinks. Pulse 140; but her skin is cold, with frequent shiverings, and her countenance very pale. Complains of pain in her limbs, and she cannot get up stairs without assistance. Her bowels have not been open for two days. She has never menstruated.

She was ordered to take a dose of Calomel with Scammony immediately; and four hours after, Infusion of Roses, with dilute Sulphuric Acid, Syrup of Poppies, Sulphate of Magnesia, and Compound Tincture of Cardamoms, of each half a drachm, to be continued three times a-day.

October 31st.—Her bowels have been acted upon only once by the medicine, and she complains of pain in them, although they are not hard nor tumid. Her mouth has become much cleaner, and there has been scarcely any bleeding since she has taken the acid draught.

A dose of Jalap and Calomel immediately; afterwards continue the draught.

November 1st.—After passing a very copious stool, of a brown colour, she felt considerably relieved.

Let the Jalap and Calomel be repeated to-morrow morning.

2d.—Her bowels have again been acted upon freely, and the stools are of a lighter colour. She feels much better, and has no pain in her limbs.

The Calomel and Jalap were again repeated on the mornings of the 3d and 5th November, and occasionally afterwards.

On the 7th, the petechiæ and ecchymoses had nearly disappeared. Pulse 120, rather sharp, and fuller than before.

Tinct. Digital. m. v. to be added to the draught.

9th.—Pulse still frequent, but not so sharp.

Mist. Camph. c. Tinct. Ferri Muriat. m. x.; & Tinct. Digital. m. v. ter die.

On the 12th, she felt considerably stronger; and, on the 22d, was discharged perfectly well, although the catamenia had not yet appeared.

II.—William Wilks, aged fourteen years, was admitted into the Middlesex Hospital, July 27th, 1826. He had been ill four days with dysenteric symptoms, accompanied with severe pain in the abdomen, increased by pressure. He had leeches twice applied on the abdomen; and had at first Calomel with Opium, and afterwards Ol. Ricini with Tinct. Opii, and Hydrarg. cum Cretâ with Pulv. Ipecac. C., until the stools became natural in consistence and appearance, except that they were always attended with a considerable discharge of blood. It was then observed that his legs and arms were covered with numerous petechiæ, and there were also broad patches of ecchymosis on the ankles and legs. His debility was at this time extreme, and one of the leech-bites on the abdomen had continued bleeding in spite of all attempts to stop it: but, upon the application of a small quantity of a powder, consisting of three parts of Calamine and one of the Nitric Oxyd of Mercury, the bleeding ceased immediately.

He had now given him an infusion of Mint and Cloves, with Sulphuric Acid. In the course of a few days, the intestinal hemorrhage had entirely ceased, the petechiæ and vibices were fast disappearing, and his strength rapidly returning. In less than a fortnight from the time of his commencing the use of the acid medicine, he was discharged from the hospital, convalescent.

*Remarks.*—If it be true that the proximate cause of purpura hæmorrhagica is a disproportion between the tone and strength of the capillary vessels, and the impetus of the blood which is circulating in them, we may conceive that such a state of the system may be produced—first, by an increase of the force of the circulation, whilst the vessels themselves remain healthy; secondly, by preternatural weakness of the superficial vessels, without increase of the impetus of the blood; thirdly, by a concurrence of increased impetus with vascular weakness.

But, when we consider how seldom this disorder is accompanied with signs of excessive arterial action,*—how much more it partakes of the nature of passive than of active hemorrhage,—that it occurs principally in persons naturally weak, or in those whose constitutions have been left in a state of debility by previous acute or chronic disorders,—and that it is attended with extreme weakness and depression of spirits, it appears reasonable to dismiss from the question the first of the three states of the system which have been supposed, and to refer all cases of this disorder to one or other of the latter two. Indeed, it seems far more probable that increased momentum of the circulating system, unattended with general weakness of the containing vessels, should produce aneurism or hemorrhage from rupture of the larger vessels, than that it should give rise to the petechiæ, ecchymoses, and general bleeding of purpura hæmorrhagica.

In fact, most cases of this disorder present unequivocal marks of venous congestion. It is possible that, in the production of the phenomena of the disease, congestion may be either a cause, an effect, or both. Obstruction to the circulation may act as a cause of sanguineous extravasation, and subsequently of vascular weakness; and, on the other hand, it seems not unlikely that congestion may be produced by a previous atony of the capillary vessels. Although, of all the viscera, the liver may be most frequently affected by derangement of the venous circulation, yet some pathologists appear to have gone too far in considering hepatic congestion as the sole cause of purpura hæmorrhagica. For disorders of other organs, as the spleen, the lungs, the heart, the brain, the uterus, may give rise to irregular accumulations of blood; and, in this disorder, deep-seated pains are felt in various parts, about the præcordia, and in the chest, the loins, and abdomen; and not merely in the epigastrium and right hypochondrium; and, although it is often attended with a constipated or irregular state of the bowels, yet at other times the functions of the intestines are natural. In a few instances, it is mentioned by Dr. Bateman that frequent syncope has occurred: in these, perhaps, the right side of the heart was the primary seat and origin of the disease.

Were hepatic congestion the sole cause of purpura hæ-

* We subjoin an example of *inflammatory* purpura.

morrhagica, it would be difficult to account for such cases as the fatal one mentioned by Bateman, which came on during a severe salivation, accidentally induced by a few grains of mercury, given in combination with opium for the cure of rheumatism. It is worthy of remark, that purpura simplex, or hæmorrhagica, will often be found to precede or follow a severe attack of rheumatism. Dr. YOUNG has also recorded a fatal case, which apparently originated from the effect of a small quantity of the pilula hydrargyri.

In some cases of this disorder, the spleen has been found to be enormously enlarged. In others, it has been obviously connected with thoracic disease. BATEMAN mentions a case in which a fleshy tumor was found in the situation of the thymus gland.

Nothing, however, is more evident than that purpura hæmorrhagica may be produced by defective uterine functions. It has frequently followed amenorrhœa, and has been carried off by a severe catamenial flooding, or has ceased on the return of the natural discharge. Dr. YEATS has related, in the Medical Transactions, (vol. v.) the case of a young woman, in whom this disorder immediately ceased on the appearance of a menstrual discharge: it was remarkable that this discharge coagulated, thereby showing that blood, and not the natural secretion, was at that time discharged from the mucous lining of the uterus.

In some persons purpura has appeared to be the consequence of an habitual hemorrhagic tendency. A morbid weakness of the capillary vessels being hereditary in certain families, must therefore be a constitutional malady. In such persons, a slight touch on the skin will sometimes produce the ecchymosis of a severe bruise; whilst the bite of a leech, or the drawing of a tooth, may be followed by a fatal hemorrhage.

If the view which has been taken of the pathology of the disorder be correct, the following are obviously the indications for treatment,—viz. to diminish the force of the circulation, and at the same time to increase the strength of the containing vessels. Hence we may perceive the impropriety of two opposite plans of treatment, which have both been recommended, and both have been followed, though confessedly without success.

Dr. WILLAN indiscriminately recommended a generous diet, the use of wine, Peruvian bark, and acids. But there is reason to believe that such a stimulant system has often increased the hemorrhage to a fatal extent.

With respect to the opposite plan, that of bleeding, it is probable that there are only two cases in which it is to be recommended: first, when the disorder is accompanied with active inflammation; secondly, when the hemorrhage is so alarming, and the congestion in some important organ (as, for example, the brain,) so apparent, that a revulsion is necessary in order to save the patient from the danger of instant destruction. In the first case, therefore, venesection is naturally requisite; in the second, it may be said to be allowable through necessity.

Probably, the best method of answering the first of the proposed indications, that of diminishing the force of the circulating system, without materially increasing vascular weakness, is by active and continued purging; a measure which is also particularly adapted for relieving that state of congestion which, in this disorder, is so common in the abdominal viscera. But, perhaps, the utility and necessity of purging have been somewhat over-rated by the advocates of the hepatic origin of purpura. In the first of the foregoing cases, purgatives were indicated by the state of the bowels and appearance of the evacuations; but, in the second case, in which the bowels were always open, and at first too much so, purgatives were unnecessary, and the cure was effected without them.

There are no medicines which appear to be so well calculated to answer the second indication—that of imparting strength to the vascular structure, as the mineral acids; for they are tonic without being stimulant, and at the same time, by their astringency, they are capable of restraining hemorrhage. In what manner the latter purpose is effected, appears to be somewhat doubtful. Dr. Paris observes, that "astringents would seem to moderate the morbidly-increased secretions of distant parts, and to restrain hemorrhage, by their corrugating influence upon the primæ viæ, which is extended by sympathetic action to the vascular fibre." But he adds, with regard to the effect of astringents on distant organs, that "it is a question whether, in many of such cases, the benefit arising from their use may not depend upon their tonic powers."

But, whether its operation be of a primary or secondary nature, it is certain that the sulphuric acid is eminently useful in restraining the hemorrhage of purpura.

The cases of this disorder which are connected with amenorrhœa, appear to call for chalybeate and emmenagogue medicines. Unless the circumstances should require an absolute forbearance from every kind of stimulant, the Tinct. Ferri Muriatis would appear to be a form well adapted for such cases; for it is both tonic and styptic, and at the same time is said to exert a specific influence on the urinary and generative organs. In the first of the foregoing cases, this medicine was serviceable, combined with Digitalis; a form which, if it were not sanctioned by use, would seem to be almost like *contra-indication.* But in this case the pulse was exceedingly rapid, and it is possible that, by restraining the pulse, digitalis may increase the tendency to absorption, and thereby facilitate the admission of the accompanying tonic into the system.

